# Probiotics-Containing Mucoadhesive Gel for Targeting the Dysbiosis Associated with Periodontal Diseases

**DOI:** 10.1155/2022/5007930

**Published:** 2022-02-27

**Authors:** Giuseppe Giannini, Irene Ragusa, Giulia Nerina Nardone, Sara Soldi, Marina Elli, Piera Valenti, Luigi Rosa, Emanuele Marra, Daniela Stoppoloni, Emilio Merlo Pich

**Affiliations:** ^1^R&D Alfasigma S.p.A., Via Pontina, Km. 30.400, 00071 Pomezia, Roma, Italy; ^2^Labomar S.p.A., Via F. Filzi 33, 31036 Istrana, Treviso, Italy; ^3^AAT–Advanced Analytical Technologies Srl, Via P. Majavacca 12, 29017 Fiorenzuola d'Arda, Piacenza, Italy; ^4^Department of Public Health and Infectious Diseases, University of Rome La Sapienza, Piazzale A. Moro 5, 00185 Rome, Italy; ^5^Takis s.r.l, Via di Castel Romano 100, 00128 Rome, Italy; ^6^R&D Alfasigma S.p.A., Via Ragazzi del '99, no. 5, 40133 Bologna, Italy

## Abstract

**Objective:**

Periodontitis is a common disorder that leads to the loss of both tooth and personal well-being, contributing to worsen the risk for metabolic and cardiovascular diseases. Recently, probiotics, characterized by rapid oral dispersion, have been topically used. Here, we present data of a mucoadhesive gel containing probiotics, capable of ensuring a slow release of bacteria to prevent and treat periodontitis.

**Methods:**

An original mucoadhesive gel (AL0005) that is anhydrous and of food grade, loaded with the blend of lactobacilli and plants' dry extracts, has been assayed.

**Results:**

The release kinetics of the bacterial mixture in different experimental models *in vitro*, including simulated saliva or physiological solutions, showed a significant and stable release for 5–8 hours. In one *in vivo* study of a mouse model of periodontitis, a locally applied mucoadhesive gel enriched with probiotic strains improved significantly the tissue pathology when compared with vehicle-exposed mice.

**Conclusions:**

Together, the results suggest that this mucoadhesive gel can be useful in the normalization of the gum bacterial flora and improvement of the tissue pathology of gum disorders.

## 1. Introduction

Dysbiosis of the subgingival plaque has been associated to the development of periodontitis. In a variable time frame, the normal bacterial flora, consisting of Gram-positive species, is progressively substituted by a pathogenic “red complex,” consisting mainly of facultative intracellular anaerobic pathogens such as *Porphyromonas gingivalis*, *Aggregatibacter actinomycetemcomitans*, *Treponema denticola*, *Tannerella forsythia,* and *Prevotella melaninogenica* [1, 2]. These bacteria eventually colonize the subgingival sites, escape the host's defense system due to their ability to enter inside host cells, and cause chronic inflammation and tissue damage. Therefore, periodontitis is a chronic multifactorial inflammatory disease associated with a dysbiotic dental plaque that results in local inflammation and the progressive destruction of the supporting structures of the teeth, namely periodontal ligament and alveolar bone. This is one of the most common diseases affecting the oral cavity [3]. According to the WHO, in Europe, severe periodontitis with rapid loss of tooth attachment (>2 mm/year) occurs in 5–20% of middle-aged adults (35–44 years) and about 40% of elderly (65–74 years) [4, 5]. Interestingly, it represents a risk factor for systemic complications, including preterm low birth weight, cardiovascular disease, and type 2 diabetes, requiring a timely treatment for reducing their incidence [6, 7].

Once periodontitis is diagnosed, the current gold standard treatment is the scaling and root planning (SRP) performed in the dentist's office. It consists in curettage and bacterial plaque removal from the gingival pockets followed by systemic or local antibiotic or anti-inflammatory prescriptions for some weeks. Among effective antimicrobial and anti-inflammatory substances, chlorhexidine has long been known. It is commonly used, although, after a prolonged use, it may lead to undesirable side effects such as the pigmentation of teeth and oral tissues [8, 9].

Recent meta-analyses are challenging the use of antibiotics, suggesting that the effects of systemic treatments should be balanced against their adverse events, the most important being the development of antibiotic resistance (MDR) to oral and nonoral microorganisms [10, 11]. Particularly, most antibiotics also affect the local gum commensal bacteria that are engaged in stopping the expansion of anaerobic pathogens in the plaque, damaging the natural antimicrobial barrier properties of the oral microbiome.

The identification of novel approaches to combat these pathogens by enhancing the natural antimicrobial barrier of the gum while respecting the oral microbiota is in progress. Promising approaches include the targeting of proresolution mediators, such as maresin 1 [12], or endothelial modulators, such as asymmetric dimethylarginine (ADMA) [13], known to play a role in the complex healing process associated to local inflammation.

For the treatment of intraosseous periodontal defects, in recent years, the results of various researches have been published. Through the use of resorbable biopolymers of hyaluronic acid, have been prepared mucoadhesive buccal films based on hydroxypropylmethylcellulose for the local release of *Levilactobacillus brevis* CD2 in order to ensure a controlled local release of enzymes, such as arginine deaminase and sphingomyelinase, which have anti-inflammatory properties [14–21].

Hydrolase produces L-citrulline and ammonia from L-arginine, with a reduction in nitric oxide levels and therefore in the levels of some of the known inflammatory cytokines. Bacterial sphingomyelinase can hydrolyze platelet activating factor (PAF), a potent inflammatory cytokine known to be associated with oral mucositis in radiotherapy. In addition to the PAF, also the ADMA, a well-known inhibitor of the metabolism of nitric oxide, is critical in some oral diseases. Actually, Isola et al. have demonstrated how serum and salivary ADMA levels increase both in periodontitis patients and coronary heart disease ones, despite ADMA not being closely related to periodontal disease severity [13].

To partially overcome these events, local delivery systems have been studied for antibiotics that were generally used as systemic treatment. For example, mucoadhesive formulations were developed to deliver metronidazole and satranidazole [22]. These formulations were based on different gelling agents, such as carboxy-cellulose, hydro-cellulose, or the Carbopol® 934P polymer, all designed to adhere to the gums for several hours or days, therefore reducing the dosing frequency and ensuring a prolonged action.

The administration of probiotics (live bacteria) as oral dispersion has also been proposed as an aid to proper therapeutics and a possible alternative to antibiotics, at least in certain circumstances. The aim of these supplementation is the enrichment of the oral microbiota with nonpathogenic commensals to colonize subgingival plaque and counteract the “red complex” bacteria expansion and to produce anti-inflammatory effects, possibly via activation of the Tool-like receptor pathways [23]. The rational of using live bacteria such as *Lactobacillus* and *Bifidobacterium* in the restoration of normal functioning of infected or inflamed tissues is based on converging experimental findings collected *in vitro* and *in vivo*, as recently reviewed by Sanders et al. [24].

In particular, randomized controlled trials were performed with probiotics oral dispersion in subjects with periodontal health problems. In one study [25], lozenges containing *Limosilactobacillus reuteri* DSM 17938 and ATC PTA 5289 improved periodontal health in navy sailors at sea when compared with placebo. Bleeding on probing, gingival index, plaque control record, probing attachment level, and probing pocket depth were improved when assessed at 14 and 42 days of treatment, showing highly significant improvements when compared with placebo. In another randomized placebo-controlled trial [26], lozenges containing *Bifidobacterium animalis* subsp*. lactis* (*B. lactis*) HN019 were used as adjuvant to scaling and root planning (SRP) standard treatment in patients with periodontitis. Forty-one patients were recruited and monitored clinically, immunologically, and microbiologically. The group receiving the probiotic lozenges showed improvement in all the monitored markers when compared with placebo at 30 and 90 days. Interestingly, other probiotics, such as *Streptococcus salivarius* K12, *Lacticaseibacillus rhamnosus* SP1, and *Lactobacillus helveticus* SBT2171, were also found to improve periodontal health [27–29].

The use of probiotics has been also investigated in prevention and treatment of cancer therapy-induced oral mucositis. A systematic review and meta-analysis based on five studies on a total of 435 patients showed the efficacy of probiotics in the reduction of incidence and/or mitigation of the severity of this side effect of cancer therapy, which is extremely detrimental for patients' quality of life [30].

Overall, these studies support the tenet that oral exposure to Gram-positive probiotics could reinstate a normal bacterial flora in the gum of subjects at risk of periodontitis and contribute to the improvement of periodontal tissue damage in subjects affected by periodontitis.

Finally, modulation of the immune system is one of the most plausible mechanisms underlying the beneficial effects of probiotics on human health. The administration of exogenous probiotics could enhance the innate immunity as well as modulate pathogen-induced inflammation via Toll-like receptor-regulated signaling pathways [31]. Moreover, the probiotic administration improves commensal barrier and, consequently, the competition against pathogenic bacteria [32]. The competition between commensals and pathogens is a principle of general validity, applicable both in the treatment of all dysbiosis including the treatment of periodontitis.

In these studies [25, 26], the lozenges allowed the exposure of the whole mouth to probiotics for a period estimated in tens of minutes/day, requiring several daily administrations. It is possible that selective local targeting of specific gum region affected by dysbiosis with high-concentration probiotics for several hours/days (instead of minutes) would hasten the normalization of the bacteria flora of the subgingival plaque. Such delivery profile could be provided by mucoadhesive preparations, which are generally well accepted by the users.

Mucoadhesive preparations consist of solid systems based on polymers that adhere to mucosae [14]. So far, among oral drug delivery methods for the treatment of periodontitis, there are mucoadhesive gel preparations releasing chlorhexidine, antibiotics, hydrogen peroxide, and natural substances [15, 16, 33]. Polymers by themselves may have an additional therapeutic effect: a recent study showed the use of a resorbable biopolymer of hyaluronic acid for the treatment of infrabony periodontal defects [34]. A proof of concept that mucoadhesive gel preparations could deliver probiotics in the oral cavity was recently published: Abruzzo et al. reported a preparation of hydroxypropyl methylcellulose mucoadhesive buccal films containing *Levilactobacillus brevis* CD2 [35].

The aim of the present work was to develop a stable formulation of a mucoadhesive lipogel that would be capable to release probiotics of the *Lactobacillus* genus, mostly known for their putative eubiotic actions and capacity to replicate in a hostile environment. The formulation was added with two natural ingredients, aloe and blueberry extracts. Nutrients found in cranberries and blueberries can be highly effective in protecting the teeth against bacteria responsible for accelerating tooth decay [36–38], and adding lemon essential oil gives the formulation a pleasant taste. The formulation is strictly anhydrous to prevent bacterial growth. The gel should be applied on the dysbiotic plaque for the restoration of the bacteria flora, eventually resulting in improvement of periodontal health. In this article we will describe the gel preparation, its characterization *in vitro*, and preclinical study in mice.

## 2. Material and Methods

### 2.1. Mucoadhesive Gel Preparation

For the preparation of the mucoadhesive gels (AL0005), cosmetic-grade ingredients compatible with the oral cavity and normally used in marketed products, such as toothpastes, were used (Table 1). To ensure the stability of the lipogel, modified silicas (MPs) such as silica dimethyl silylate (AEROSIL) and hydroxypropyl methylcellulose (HPMC) (BENECEL K100M) were also used, as described in Table 1.

### 2.2. Bacteria under Study

A series of *Lactobacillus* strains with known antimicrobial activity and mucosal colony-forming properties have been considered: *Levilactobacillus brevis (L. brevis)*, *Lactiplantibacillus plantarum (L. plantarum)*, *Lacticaseibacillus paracasei (L. paracasei)*, *Limosilactobacillus reuteri (L. reuteri)*, and *Lacticaseibacillus rhamnosus (L. rhamnosus)*, so defined according to a recent nomenclature [39]. These probiotics were kept in lyophilized form until blending into lipogel.

### 2.3. Determination of Bacterial Load

To estimate the total bacterial load, both in the lyophilized powder (starting material) and in lipogels, as well as their relative release from the lipogels, at different times, the classic method of colony-forming unit (CFU) count and an innovative method, named Lacto-Counter Assay (LCA), were applied [40, 41].

CFU count is an internationally validated and applied method. However, it is not reliable if the bacteria are aggregated or in biofilm. LCA is a specific assay for the enumeration of lactobacilli based on the BioTimer Assay (BTA), a biological, innovative method that allows us to count free, aggregated, adherent, or biofilm microorganisms without manipulating the sample [40, 41]. In these tests, to enumerate *Lactobacillus* spp., an original reagent that contains a pH indicator able to change color related to the microbial metabolism (production of secondary metabolites) was developed. The concentration of secondary metabolites was correlated to the number of bacteria present in the sample at time 0 by means of calibration curves, reported in the following paragraph.

#### 2.3.1. LCA Specific Reactive to Count *Lactobacillus* spp

**Figure 1:**
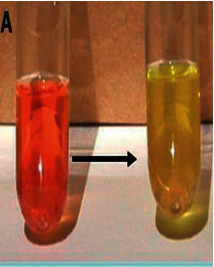
Lacto-Counter Assay (LCA) reagent with the phenol-red shifts the color from red to yellow due to its acidification by the lactobacilli metabolism.

The LCA specific reactive was prepared as follows: 5.2 g of “de Man, Rogosa, and Sharpe” (MRS) broth (Oxoid LTD, UK) was dissolved in 810 ml of distilled water. After sterilization at 121°C for 15 min, 80 ml of 10% filtered glucose (Sigma Aldrich, Italy) solution, 100 ml of 10% filtered lactose (Sigma Aldrich, Italy), and 10 ml of 0.25% filtered phenol red (Sigma Aldrich, Italy) solution were added and the pH was adjusted to 7.2 ± 0.1. The final reagent appeared clear and red. To perform the LCA, test tubes, containing the LCA reagent and the sample, were covered with paraffin to obtain anaerobiosis and incubated at 37°C. The color changes from red to yellow (Figure 1).

**Figure 2:**
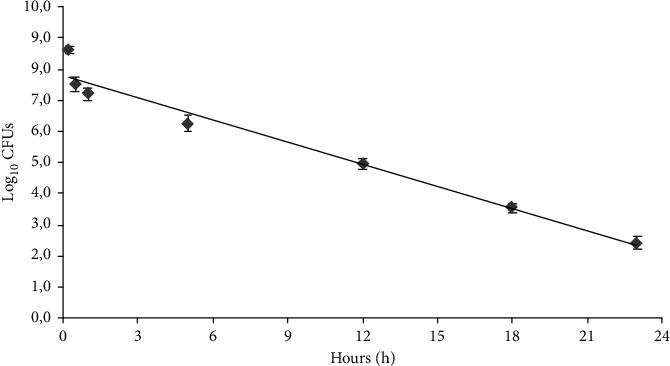
Correlation line between the number of *Lactobacillus* spp. and the time of color change of the Lacto-Counter Assay (LCA) reagent.

Color change, due to metabolism of *Lactobacillus* spp., was measured through the change of OD values at 420 nm (yellow) at regular time intervals (30 min) until 24 h. The time required for color change of LCA reagent from red to yellow was correlated to initial microbial concentration by correlation line (Figure 2). This correlation line is identical to that obtained to enumerate *Bifidobacterium* spp.

Briefly, serial twofold dilutions of distinct planktonic overnight broth cultures of *Lactobacillus* spp. were performed in 24-well plates (BD, Italy) at 37°C in a total volume of 1 ml of LCA reagent and simultaneously plated in MRS agar plates and enumerated by CFU count. The time required for color switch of LCA reagent was plotted versus the mean values of the log_10_ of CFUs/ml for *Lactobacillus* spp. (Figure 2). The equation and the linear correlation coefficient describing the correlation line was *y* = −0.2397*x* + 7.828*R*^2^ = 0.9646.

### 2.4. Enumeration of Lyophilized Lactobacilli by LCA and CFU Method

First, the bacteria present in the lyophilized cultures, used for lipogel formulations, were enumerated by both LCA and CFU method. Each lyophilized culture (1 g) was dissolved in LCA reagent (10 ml) to have a final concentration of 100 mg/ml. This sample was incubated at 37°C in anaerobiosis, monitoring over time the color change of LCA reagent. In parallel, 100 *μ*l of this solution, after appropriate dilutions, was plated in MRS agar plates and enumerated by CFU count.

### 2.5. Test of Bacteria Release in Physiological Solution or Simulated Saliva

Lipogel preparations containing *Lactobacillus* strains were profiled for their release properties when immersed in a liquid phase consisting either of physiological solution (solution) or simulated saliva (SS) prepared according to Marques et al. [42]. SS composition corresponded to sodium chloride (8 g/L), sodium phosphate dibasic (2.38 g/L), and potassium phosphate monobasic (0.19 g/l) with a pH of 6.8. Briefly, at time 0, samples of lipogels were immersed in LCA and tested to evaluate the total bacterial load at baseline. Subsequently, samples of lipogels were immersed in the solution/SS and bacterial release was evaluated after 30 min, 2, 5, and 8 h. At each incubation time, 1 ml of solution was collected and mixed with 1 ml of LCA reagent to quantify the number of bacteria released. In parallel, to check the bacteria still present in the lipogels at each time, an aliquot of lipogel was collected, weighted, and immersed in 1 ml of LCA. The number of loaded or released bacteria were normalized at 1 g of lipogel. The loaded bacteria in the lipogel were enumerated only with LCA because CFU count was considered unreliable in the counting bacteria inside the lipogel. The bacteria released in solution/SS were enumerated using both LCA and CFU count.

### 2.6. Test of Adhesion and Bacteria Release on Sterile Plastic Cones

**Figure 3:**
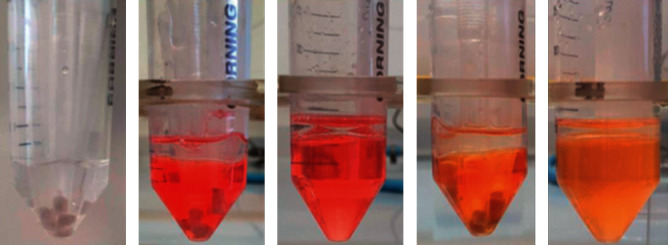
Representative picture of the model developed: (a) plastic cones with the mucoadhesive lipogel incorporated immersed in artificial saliva; (b) plastic cones with the mucoadhesive lipogel incorporated immersed in Lacto-Counter Assay (LCA) reagent after 2 h of incubation with artificial saliva; (c) artificial saliva immerged in LCA reagent after 2 h of incubation with plastic cones containing the mucoadhesive lipogel incorporated; (d) color change of sample B solution in LCA; and (e) color change of sample C solution in LCA.

To simulate the conditions of the lipogel application on a gingival surface of the mouth, a model based on plastic cones containing the mucoadhesive lipogels was developed (Figure 3). Such cones were then immersed in different solutions (i.e., physiological solution, PBS, artificial saliva) and incubated at 37°C. Samples of the solutions were collected at different time points and checked for the bacterial amount released that was enumerated using both LCA and CFUs.

### 2.7. Test of Bacteria Release on Filter Papers

A further qualitative test was performed using filter papers to evaluate the release of lactobacilli from lipogels. Briefly, 1 g of each lipogel was diluted in 99 ml of MRD (maximum recovery diluent) or SS (simulated saliva) and serially diluted. Sterilized filter papers were kept in contact respectively for 2 and 5 hours with diluted lipogels. Filter papers were recovered with sterile forceps and placed on MRS agar plates and incubated for 72 h at 37°C under anaerobic conditions. Colonies released and adhered on filter papers were counted and compared with those obtained at the same dilution for quantitative enumerations.

### 2.8. Test of Adhesion to Hydroxyapatite Discs

To compare the adhesive properties of bacteria released from lipogels with the same capability of the whole lipogel, we optimized a model using 100% synthetic tri-calcium phosphate-based 3D Biotek *β*-TCP discs. This assay was performed according to Tan et al. [43] with minor modifications. Each lipogel (1 g) was diluted in 99 ml of MRD or in 99 ml of SS, until a concentration of about 10^6^ CFUs/ml was reached. 1 ml of the four suspensions was used for viable count determination (T0 time point), and an equal aliquot was used to cover 3D Biotek *β*-TCP models for 2 and 5 hours. Following incubation, 3D Biotek *β*-TCP discs were collected in a 9 ml tube of diluent and bacteria retained on the surface were removed by mild ultrasonication and rapid vortex mixing. The number of total bacteria was determined by means of viable counts, and the percentage of adhesion calculated as follow:

(1) $$\begin{array}{c} P=\frac{\mu }{M}*100, \end{array}$$

where (i) *P* represents the adhesion percentage of analyzed strains to 3D Biotek *β*-TCP discs; (ii) *μ* represents the viable count of analyzed lipogels bonded to 3D Biotek *β*-TCP discs expressed as a logarithmic value; and (iii) *M* represents the viable count of analyzed lipogels transformed as a logarithmic value of the T0 suspension total charge.

### 2.9. Antimicrobial Activity: Competition Test

To assess the ability of *Lactobacillus* released by the lipogel preparations to counteract the presence and adhesion of different strains of oral pathogens, a competition test was performed according to Shokryazdan et al. [44]. Five different strains present in the “red complex” were selected: *Porphyromonas gingivalis* DSM 20709, *Treponema denticola* DSM 14222, *Tannerella forsythia* ATCC 43037*, Prevotella melaninogenica* DSM 7089, and *Aggregatibacter actinomycetemcomitans* ATCC 700685. The pathogens were purchased from international bacterial collection and cultivated on Columbia blood agar base (Oxoid, Thermo Fisher Scientific) supplemented with 5% defibrinated horse blood. To allow the growth of *T. forsythia* (ATCC 43037), N-acetylmuramic acid (NAMA; Sigma-Aldrich) was added to the medium at a final concentration of 10 *μ*g/ml. Columbia medium was prepared accordingly to the manufacturer's instructions and was sterilized at 121°C for 15 minutes. The medium was thawed in a water bath and directly poured in plates within an anaerobic chamber (atmosphere 85% N_2_, 10% CO_2_, 5% H_2_). Strains were revitalized in the anaerobic cabinet by direct streak on oxygen-free Columbia blood agar plates. Plates were incubated for 48 h at 37°C under anaerobic atmosphere. The halo size produced by each strain against each pathogen was measured in duplicate.

### 2.10. Antimicrobial Activity: Agar Well Diffusion Test

For the assessment of the antagonistic activities of the Lactobacillus strains against the 5 pathogens, the approaches described by Shokryazdan et al. [44], Tourè et al. [45], and Sajedinejad et al. [46] were considered. In order to avoid possible problems due to the mixing and spreading of different bacterial cultures on the same plate surface, modifications to the methods were applied. Six-well culture plates were poured in the anaerobic chamber with 4 ml of Columbia blood agar (5%) and allowed to solidify. *P. melaninogenica* DSM 7089, *P. gingivalis* DSM 20709, *T. denticola* DSM 14222, *T. forsythia* ATCC 43037, and *A. actinomycetemcomitans* ATCC 700685 were cultured in Brucella broth (Liofilchem) supplemented with 5% of horse blood for 48 h at 37°C in an oxygen-free cabinet. Pathogens were inoculated with a cotton swab on Columbia blood agar microplates, and the wells for diffusion assay were prepared using a sterile cork borer. Lipogel preparations were accurately weighted and diluted 1 : 20 with artificial saliva (SS) [42]. Liquid cultures of the *Lactobacillus* blend were centrifuged 10 minutes at 3000 rpm; the supernatants were collected and divided in two aliquots. The pH was measured for one of the two aliquots, while the other was adjusted to 7 with NaOH 1 M. All supernatants were 0.22 *μ*m filter-sterilized. Chlorhexidine digluconate aqueous solution (Sigma-Aldrich) was diluted at 0.2% concentration, and 100 *μ*l of the prepared solutions were dispensed in the inoculated wells to provide antibacterial control. Plates were incubated at 37°C for 48 h in the anaerobic chamber.

### 2.11. Ligature-Induced Periodontitis in C57Bl/6 Mice

Rodents are the ideal animal model for the pathological and therapeutic research of periodontitis, among these in particular the mouse models are the most convenient and versatile.

In recent years, several models have been developed by injection of human oral bacteria or chemicals or by placing a retentive ligature around the molar teeth. The ligature-induced periodontitis model has several advantages compared with other models, including rapid disease induction and predictable bone loss that can be easily measured and quantified. In this model, a ligature is placed around the teeth to facilitate development of a dysbiotic oral microbiota and damage to the gingival tissue. Indeed, the ligatures are able to facilitate local accumulation of bacteria and thereby enhance bacteria-mediated inflammation and bone loss [47–50].

The in vivo effect of lipogel preparations was assessed using the mouse model described by Marchesan et al. [49] and Abe and Hajishengallis [47] with modifications. At day 0, mice are lightly anaesthetized with the combination of tiletamine and xylazine (25 mg/kg and 4 mg/kg, respectively), and the maxillary second molar tooth is ligated by a 5-0 nonabsorbable silk surgical suture to induce periodontal inflammation. Treatments are applied daily (see paragraphs 3.5 and 4.5). Animals are sacrificed by CO_2_ asphyxia at different time points postligation. The contralateral molar tooth unligated served as baseline control for bone height measurements. To obtain such measurements, after the sacrifice, skulls are dissected out and boiled in water at 15 psi for 10 min. After defleshing, the skulls are subjected to brushing and bleaching. The maxillae are stained with a 0.5% eosin and 1% methylene solution. Periodontal bone heights are assessed using a stereomicroscope connected to a Nikon Digital Sight camera and measured using Zen software. Measurements are performed on the second molar (buccal groove). Periodontal bone heights are measured as the distances from the cementoenamel junction (CEJ) to the alveolar bone crest (ABC). The total bone loss in the ligated side was calculated subtracting the CEJ-ABC distance for the ligated side of each mouse from the CEJ-ABC distance of the contralateral unligated side of the same animal.

## 3. Experimental Plan

### 3.1. Identification of the Lactobacillus Strains to be Included in the Lipogel Preparations

In a preliminary experiment, various preparations of lipogel were loaded individually with either *Bifidobacterium animalis* subsp. *Lactis* BLC1 (DSM17741), or *L. reuteri* LR92 (DSM 26866), or *L. brevis* SP48 (DSM 16806), or *L. helveticus* SP27 (DSM29575), or *L. rhamnosus* SP1 (DSM21690), or *L. paracasei* LMG-S-26420 (CBA-L87), obtaining 6 different preparations. The goal of this experiment was to identify at least 3 strains able to remain viable into the lipogel preparations and they were released into the liquid solutions for at least 8 hours, reaching at that time the same bacteria enumeration measured in the respective lipogel.

### 3.2. Development of Two Lipogel Preparations (AL0006 and AL0007) with Different Blends of *Lactobacillus* Strains and Assessment of Their Performance

Two different lipogel preparations containing 2 and 3 different *Lactobacillus* strains were selected and prepared in two different batches: the first one on a 10-gram scale, with a lower quantity of bacteria (2 × 10(8) CFU/g), and the second one on a 100-gram scale, with a higher quantity of bacteria (2 × 10(10) CFU/g). These 2 lipogel preparations were assessed in a series of studies aimed to optimize the lipogel formulations in respect to their proposed use in human periodontal mucosa, focusing on adhesive properties and bacterial release profiles. The tests were (a) adhesion and release on sterile plastic cones, (b) bacterial release on filter papers, and (c) adhesion to hydroxyapatite discs.

### 3.3. Stability of Lactobacillus in the AL0006 and AL0007 Lipogel Preparations over Time

The stability and the bacterial release ability of the two formulations (AL0006 and AL0007) when maintained at room temperature (21°C) were monitored after 3 (T3), 6 (T6), 9 (T9), and 12 (T12) months with the LCA technique in order to overcome the limits of the traditional CFU counts. At each time point, the release of the different components of the lactobacilli blend was assessed in a physiological solution up to 5 h. The loaded bacteria in the lipogel were enumerated with LCA, while the bacteria released were enumerated using both LCA and CFUs.

### 3.4. Assessment of the Antimicrobial Activity against “Red Complex” Pathogenic Strains

The competition test (paragraphs 2.8; 2.9) was run on various lipogel preparations containing single probiotics *L. helveticus* SP27 or *L. rhamnosus* SP1 or *L. paracasei* LMG-S-26420 (CBA-L87) against *P. melaninogenica* DSM 7089, *P. gingivalis* DSM 20709, *T. denticola* DSM 14222, *T. forsythia* ATCC 43037, and *A. actinomycetemcomitans* ATCC 700685, respectively. Strains were revitalized in the anaerobic cabinet by direct streak on oxygen-free Columbia blood agar plates. Plates were incubated for 48 h at 37°C under an anaerobic atmosphere. The halo size produced by each strain against each pathogen was measured in duplicate.

Eleven different conditions were tested in duplicate against the pathogens and their blend to observe their antimicrobial activities. *P. melaninogenica* DSM 7089, *P. gingivalis* DSM 20709, *T. denticola* DSM 14222, *T. forsythia* ATCC 43037, and *A. actinomycetemcomitans* ATCC 700685 or all pathogen together were considered as a separate testing group. After inoculation on the Columbia blood agar microplates, diluted supernatant of centrifuged lipogel preparations either AL0006 or AL0007 was added to the wells for diffusion assay. Sterile MRS and one uncut inoculated well were used as positive growth controls, while chlorhexidine digluconate aqueous solution (Sigma-Aldrich) diluted at 0.2% was used as the positive antimicrobial control.

### 3.5. Testing Lipogel Preparation on Ligature-Induced Experimental Periodontitis in C57Bl/6 Mice

The ligature-induced periodontitis model was performed as described in the Methods section. C57Bl/6 mice, male, 9 weeks old with a body weight between 18 and 20 g upon arrival, were used for this study. Mice were divided into three experimental groups (15 mice/group): the first group exposed to AL0006 (active treatment), the second group daily exposed to AL0005 (lipogel alone), and the third group left untreated. From day 1, the mice of groups 1–2 were treated under light anaesthesia (isoflurane) with the group-specific lipogel, daily from Monday to Friday for 3 weeks. The gels were applied late in the afternoon, and the animals were maintained without drinking for 1 h at least and fasted overnight. The animals (3 mice/group) were sacrificed by CO_2_ asphyxia at three different time points postligation (days 8, 15, and 22). The remaining mice were sacrificed at day 29. The contralateral molar tooth unligated served as baseline control for bone height measurements. Animal studies were performed in accordance with the European Directive 2010/63/EU on the protection of animals used for scientific purposes, applied in Italy by the Legislative Decree 4 March 2014, n. 26.

### 3.6. Statistical Analysis

Results were expressed as the mean values ± standard deviation (SD) of three independent experiments. For inferential purpose, ANOVA or Kruskal Wallis was used, followed by post hoc comparison tests. In each case, a *p* value ≤ 0.05 was considered statistically significant.

## 4. Results

### 4.1. Viable Bacterial Load and Release from Lipogels

**Figure 4:**
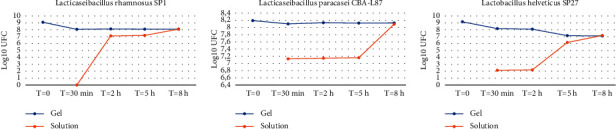
Enumeration of viable *Lactobacillus* from various lipogel preparations. Measurements were performed with LCA for the bacteria within the lipogels (blue line) and with both LCA and plate-spreading in agar for the bacteria released in solutions (*Y*-axis) at different time points (*X*-axis). Gel: lipogel preparations loaded with bacteria. Solution: artificial saliva was prepared according to Marques et al. [42]. Values are expressed as mean of three independent experiments.

The results related to the preliminary experiments are reported in Figure 4. Out of 6 different lipogel preparations with different lactobacilli, those containing *L. rhamnosus* SP1, *L. helveticus* SP27, and *L. paracasei* CBAL87 were selected, given their stable presence of viable bacteria in the lipogel over time (blue line) and their level of release of viable bacteria in a physiological solution for up to 8 hours (red line). The data obtained with the other lactobacilli initially considered are not reported here.

### 4.2. Development of Two Lipogel Preparations (AL0006 and AL0007)

Three strains of *Lactobacillus* selected since they fitted the minimal requirements obtained in the preliminary experiments, i.e., *L. rhamnosus* SP1, *L. helveticus* SP27, and *L. paracasei* CBAL87 were considered for proposing the two lipogel preparations carrying a mixture of *Lactobacillus:* the first, named AL0006, containing 2 strains, i.e., *L. rhamnosus* SP1 and *L. helveticus* SP27, and the second, named AL0007, containing 3 strains, i.e., *L. rhamnosus* SP1, *L. helveticus* SP27, and *L. paracasei* CBAL87. Details of the bacteria enumeration in the high-load lipogel preparations are shown in Table 2.

#### 4.2.1. Bacteria Release in Solution/SS over Time

**Figure 5:**
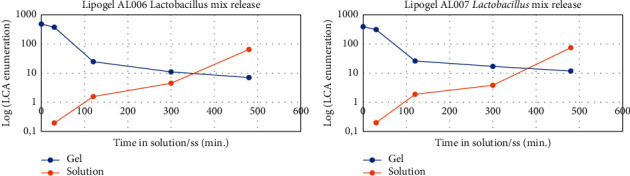
Lacto-Counter Assay (LCA) enumeration of *Lactobacillus* mix contained in lipogels AL0006 and AL0007 and released in physiological solution. Time T0 is referred to the quantity of lactobacilli present in the lipogels, while time points 30, 120, 300, and 480 min refer to the quantity of bacteria remaining in the lipogels (1 g) or released in physiological solution (solution) (1 g/ml) after each incubation time.

The performance of the two Lipogel preparations with *Lactobacillus* mixture AL0006 and AL0007 was studied by assessing changes in the bacterial load in each lipogel over time, i.e., the number of bacteria released in a physiological solution (g/ml) and the residual bacteria in the lipogel (g) were measured at various intervals up to 8 h (Figure 5). AL0006 was loaded with 1.3 × 10^9^ CFU/g of *L. rhamnosus* SP1 (1%) and 4.1 × 10^9^ CFU/g of *L. helveticus* SP27 (1%), while AL0007 was loaded with 8.71 × 10^8^ CFU/g *L. rhamnosus* SP1, 2.75 × 10^9^ CFU/g *L. helveticus* SP27, and 2.72 × 10^7^ CFU/g *L. paracasei* CBAL87.

#### 4.2.2. Bacteria Release on Filter Papers

**Figure 6:**
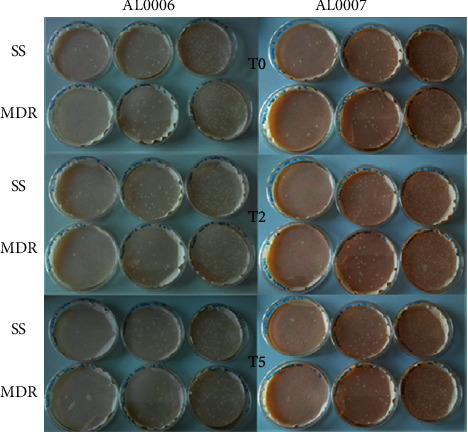
Comparison between the release of bacteria from filter papers added with the two lipogel preparations AL0006 and AL0007 in presence of MRD or SS and measured at different time points (T0: baseline, T2: 2 h, T5: 5 h).

The two lipogels AL0006 and AL0007 were spread on filter paper disks, separately, and immersed in either MRD or SS. Bacteria were then counted, with the aim to provide an estimated number of bacteria released over time in the two solutions. As shown in Figure 6, both lipogel preparations released bacteria in presence of both diluents. Semiquantitative results on filter paper were plotted against quantitative measurements on MRS plates at the same three different time points in presence of both diluents and are presented in Table 3. The table shows the percentage of recovery of released bacteria on the surface of filter paper. Percentages were calculated by considering 100% the logarithmic count obtained by the quantitative method divided by the log-transformed value of colonies recovered on paper. The result of the semiquantitative filter test agreed with those obtained with the plate-spreading on the MRS agar test. MRD allowed a slightly higher recovery on filter paper compared with SS, probably due to the presence of a small percentage of peptone in its recipe. Overall, in MRD or SS, both gels, AL0006 and AL0007, released bacteria in the same range.

#### 4.2.3. Test of Adhesion to Hydroxyapatite Discs

The Synthetic tricalcium phosphate/hydroxyapatite (TCP-HA) disc model has been used to evaluate the adhesive properties of the gels and the bacteria released according to Tan et al. [43] with minor modifications as described in the Method section. As shown in Table 4, lipogel AL0006 seemed to be fostered in adhesion in MRD, compared to SS, however such gap was reduced by observing the adhesion after 5 hours, due an enhancing the percentage of adhesion in SS.

Conversely, AL0007 displayed a higher percentage of adhesion after 5-h incubation in presence of SS than with MRD, with a significant increase in the release of viable bacteria from 2- to 5-hour coincubation. This gel showed in the normal laboratory diluent a reduction in the percentage of adhesion following coincubation from 2 to 5 hours. The difference in adhesion of released bacteria to hydroxyapatite disks could be due to their different strain composition.

### 4.3. Stability of Bacteria Viability in Lipogels and of Their Release Performance

*Lactobacillus* viability in the two lipogel preparations AL0006 and AL0007 was assessed by means of LCA at T0 and after 3 (T3), 6 (T6), 9 (T9), and 12 (T12) months, left on a shelf at room temperature. The results of T0 are presented in Table 5. The bacterial load of the two lipogels was stable up to 6 months (T6), while a slight decrease in viability was registered for both lipogels at 9 (T9) and 12 (T12) months.

In addition, a bacteria release test over 8 h was performed at each of the stability time points (T0), (T3), (T6), (T9), and (T12) for both preparations. Results, shown in Table 5, indicated that bacterial enumerations were slightly reduced from T0 to T12, but it was interesting to observe a reproducible behaviour of both lipogel preparations in releasing the bacteria in solutions from 30 min to 8 h of incubation. This trend was evident throughout the 12 months of assessment (Table 6).

### 4.4. Assessment of the Antimicrobial Activity against “Red Complex” Pathogenic Strains

The results of the competition tests (inhibition zone) using each *Lactobacillus* strain selected for lipogel preparations against the various “red complex” pathogens are summarized in Table 7. Two strains, *L. rhamnosus* and *L. paracasei*, showed a good ability (larger inhibition zone) in counteracting the growth of all pathogens, with a numerical major activity of *L. paracasei* with respect to *L. rhamnosus*. Conversely, *L. helveticus* was not able (smaller inhibition zone) to contrast the growth of most strains, with the exception on *A. actinomycetemcomitans*.

The results of the agar well diffusion test are reported in Table 8. The antibacterial agent chlorhexidine showed a strong effect, generating wide halos in all the wells, providing an internal standard for the test. An additional treatment group was included, containing the lipogel without any bacteria, identified as AL0005. Among the bacteria-loaded lipogel preparations, AL0006 gel was the most effective since it was the only preparation active towards the blend of all the pathogenic strains. Conversely, AL0007 gel was not effective towards the all-pathogen blend but exerted a stronger effect on *A. actinomycetemcomitans*, probably due to the presence of *L. paracasei*. However, the presence of *L. paracasei* may affect the performance of *L. rhamnosus* and *L. helveticus,* the two strains present in AL0006. Intriguingly, the lipogel formulation without bacteria AL0005 showed by itself a good activity against the single strains of *P. gingivalis*, *T. denticola*, and *T forsythia*, but not on the all-pathogen blend, probably due to the presence of botanical extracts used as ingredients in the formulation (AL0005), as described in Table 1.

### 4.5. Testing Lipogel Preparations on Ligature-Induced Experimental Periodontitis in C57Bl/6 Mice

A mouse “ligature-induced model” [47] was assessed to evaluate the efficacy of the mucoadhesive gel loaded with probiotics (AL0006) versus the same gel without probiotics (AL0005) in counteracting the progression of periodontitis.

**Figure 7:**
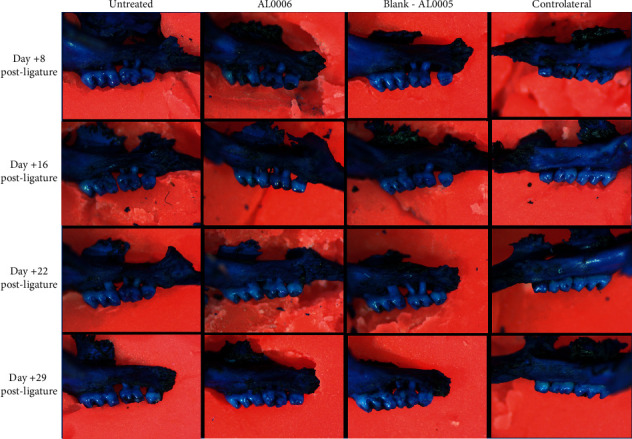
Representative images of maxillae, stained with eosin and methylene solution, at day +8, +16, +22, and +29.

As shown in Figure 7, at day +8, the treatment seemed to have no effect. In fact, the CEJ-ABC distance for the ligated side of the second molar displays significantly increased bone heights, therefore progressive bone loss in all experimental groups, markedly more evident for gel blank (AL0005)-treated groups. From day +15, the gel AL0006 treatment is shown to be able to statistically significantly counteract the progression of the bone loss compared with untreated and gel blank groups. The effectiveness of gingival gel AL0006 remains constant even after the stop of the treatments (21 days postligation) until the end of the study. The protective effect of gel AL0006 was also demonstrated by the absence of tooth loss due to less destruction of the alveolar bone and connective tissues that surround and support the teeth, a feature found only in the gel AL0006-treated group.

To further confirm the efficacy of the AL0006 gel, the data on tooth loss after processing of the skull must be considered.

Overall, after four weeks of study, in the different experimental groups of postligation mice, the loss of second molar tooth ligated was observed in 6/15 mice in the untreated group, as well as in the blank group treated with gel AL0005, while 0/15 was observed in the group treated with gel AL0006.

The effect of gingival gel treatment (gel AL0006) on the kinetics of total bone loss in the ligated buccal side versus contralateral unligated side, at 8, 15, 22, and 29 days after the placement of ligatures on the left maxillary molars in mice, is shown in S.I.  Figure 1.

## 5. Discussion

Out of 6 different lipogel preparations, with different lactobacilli, those containing *L. rhamnosus* SP1, *L. helveticus* SP27, and *L. paracasei* CBAL87 were selected given their stable presence of viable bacteria in the lipogel over time and because of their level of release of viable bacteria in a physiological solution for up to 8 hours (4.1). AL0006 (*L. rhamnosus* SP1 and *L. helveticus* SP27) and AL0007 (plus *L. paracasei* CBAL87) lipogels were prepared (4.2) and evaluated for their ability to release the included viable bacteria in different conditions (4.2.1–4.2.3).

The first experimental setup was optimized to evaluate the decimal counts of viable bacteria following simple rehydration in the ratio 1 : 100 of samples with simulated saliva (SS). Both samples revealed a very good performance by releasing 5 × 10^9^ CFUs/g or more viable bacteria following 2 hours rehydration. Prolongation of the incubation time results in a gradual increase in viable relieved cells, estimated for up to 5 hours (4.2.1).

When 1 : 100 diluted lipogels (following dilution in MRD and in SS and incubation for 2 and 5 hours) were layered on MRS Petri dishes covered with filter paper (and then incubated for 72 hours at 37°C under anaerobic conditions), both samples released bacteria over time, as shown by the clear presence of colonies on filters. (4.2.2)

Finally, when the samples were tested by means of adhesivity model, they showed good adhesive properties on synthetic tricalcium phosphate/hydroxyapatite (TCP-HA) disks. (4.2.3)

The contribution of simulated saliva to the release of viable bacteria seemed stronger over time since after 2 and 5 hours for AL0006 percentages of adhesion, in SS, increased from 62.1 to 65.3% and for AL0007 from 67 to 75.2, while values in MRD were stable for AL0006 (72.2 vs 73.0) and were reduced for AL0007 (from 70.2% to 65.8%).

Stability of bacteria viability in lipogels and of their release performance were evaluated for both lipogels AL0006 and AL0007 by means of LCA at T0 and after 3 (T3), 6 (T6), 9 (T9), and 12 (T12) months, left on a shelf at room temperature. The bacterial load of two lipogels was stable up to 6 months (T6), while a slight decrease in viability was registered for both lipogels at 9 (T9) and 12 (T12) months. It was interesting to observe a reproducible behaviour of both lipogel preparations in releasing the bacteria in solutions from 30 min to 8 h of incubation. This trend was evident throughout the 12 months of assessment (4.3).

This study was completed with an assessment of the antimicrobial activity of two lipogels against “red complex” pathogenic strains, versus the same lipogel without bacteria (AL0005), and an in vivo test on ligature-induced experimental periodontitis in C57Bl/6 mice on the mucoadhesive gel loaded with probiotics (AL0006) versus the same gel without probiotics (AL0005).

In general, the results of the competition tests showed a good ability of two-strain formulation (AL0006) in counteracting the growth of all pathogens. It was more effective than AL0007 gel and the corresponding bacteria-free lipogel formulation (AL0005). AL0007 gel was less effective towards pathogenic blend and *P. melaninogenica* but exerted a stronger effect on *A. actinomycetemcomitans*, probably due to the positive interaction of probiotic strains within the mixture (4.4).

Lastly, a ligature-induced periodontitis mouse model was assessed to evaluate the efficacy of the mucoadhesive gel loaded with probiotics (AL0006) versus the same gel without probiotics (AL0005) in counteracting the progression of periodontitis. Evaluated over four weeks, the last without gel application, after a week the cementoenamel junction alveolar bone crest (CEJ-ABC) distance for the ligated side of the second molar displays significantly increased bone heights, therefore progressive bone loss in all experimental groups, markedly more evident for gel blank (AL0005)-treated groups. From day +15, the gel AL0006 treatment is shown to be able to statistically significantly counteract the progression of the bone loss compared with untreated and gel blank groups. The effectiveness of gingival gel AL0006 remains constant even after the stop of the treatments (21 days postligation) until the end of the study. The protective effect of gel AL0006 was also demonstrated by the absence of tooth loss due to less destruction of the alveolar bone and connective tissues that surround and support the teeth, a feature found only in the gel AL0006-treated group. (5)

## 6. Conclusion

Dysbiosis of the subgingival plaque has been associated to the development of periodontitis where the normal bacterial flora is progressively substituted by a pathogenic “red complex,” consisting mainly of anaerobic facultative intracellular pathogens such as *Porphyromonas gingivalis*, *Aggregatibacter actinomycetemcomitans*, *Treponema denticola*, *Tannerella forsythia,* and *Prevotella melaninogenica*. These bacteria eventually colonize the subgingival sites, escape the host's defense system due to their ability to enter inside host cells, and cause chronic inflammation and tissue damage. Therefore, periodontitis can be associated with a chronic, multifactorial inflammatory form caused by oral dysbiosis. These events result in progressive destruction of the supporting structures of the teeth, namely periodontal ligament and alveolar bone, one of the most common diseases affecting the oral cavity. Interestingly, these events should not be considered as only a disorder affecting the oral cavity normally associated, in the most serious cases, with tooth loss, but its effects also spread at a systemic level with a negative impact on metabolism (i.e., type 2 diabetes), pregnancy (preterm low birth weight), cardiovascular system, and many others.

Once periodontitis is diagnosed, the current gold standard treatment is the scaling and root planning (SRP) consisting in curettage and bacterial plaque removal from the gingival pockets followed by systemic antibiotic and anti-inflammatory prescriptions for some weeks, with the risk of also altering the natural oral microbiota.

For the treatment of intraosseous periodontal defects, in recent years the results of various researches have been published. Through the use of resorbable biopolymers of hyaluronic acid have been prepared mucoadhesive buccal films based on hydroxypropylmethylcellulose (HPMC) for the local release of Levilactobacillus brevis CD2 in order to ensure a controlled local release of enzymes, such as arginine deaminase and sphingomyelinase, which have anti-inflammatory properties. Hydrolase produces L-citrulline and ammonia from L-arginine, with a reduction in nitric oxide levels and therefore in the levels of some of the known inflammatory cytokines. Bacterial sphingomyelinase can hydrolyze platelet activating factor (PAF), a potent inflammatory cytokine known to be associated with oral mucositis in radiotherapy. In addition to the PAF, also the asymmetric dimethylarginine (ADMA), a well-known inhibitor of the metabolism of nitric oxide, is critical in some oral diseases as recently demonstrated, where higher serum and salivary ADMA levels increase both in periodontitis patients and coronary heart disease ones, despite ADMA not being closely related to periodontal disease severity.

To partially overcome these events, local delivery systems have been studied. For example, mucoadhesive formulations were developed to deliver antibiotics. These formulations were based on different gelling agents, such as carboxy-cellulose, hydro-cellulose, or the Carbopol® 934P polymer, all designed to adhere to the gums for several hours or days, therefore reducing the dosing frequency and ensuring a prolonged action.

Mucoadhesive-based gel films are considered suitable formulations in terms of comfort, and there are few biomaterials that can be used for this purpose, such as modified cellulose and hyaluronic acid.

Through a systematic preliminary investigation, we have now assessed the load, survival, and release of a set of bacterial strains among those described in literature to have a positive impact on oral dysbiosis. The mucoadhesive gel formulations thus prepared, enriched with single strains, were left up to eight hours in a physiological solution or in artificial saliva. After an initial screening, three probiotics were selected and two different formulations were prepared, the first one including *Lactobacillus helveticus SP27* and *Lacticaseibacillus rhamnosus SP1* (AL0006) and the second one that was further enriched with *Lacticaseibacillus paracasei CBA-L87* (AL0007). In the viability, stability, and in vitro release tests, these bacterial consortia confirmed the results initially obtained on individual strains. And they were subsequently evaluated in an in vivo study in a mouse periodontitis model.

The lipogel (AL0005) was prepared with caprylic/capric triglyceride, ethylcellulose, and hydroxypropyl methylcellulose, glyceryl behenate, and silica dimethyl silylate. The formulation was added with two natural ingredients, aloe and blueberry extracts. Nutrients in cranberries and blueberries can be highly effective in protecting the teeth against a complex of bacteria responsible for accelerating tooth decay, and adding lemon essential oil gives the formulation a pleasant taste. The formulation is strictly anhydrous to prevent bacterial growth.

Overall, the aim of the project was to develop a mucoadhesive gel (lipogel) film able to steadily deliver a consortium (two or more) of probiotics in the mouth cavity, over a period of 5–8 hours, compatible with an evening application before going to sleep, ensuring a slow release during the night. Furthermore, the formulation should ensure stability over time, compatible with product commercialization. The synergistic effect of probiotics and botanicals represents a novelty in this field for the benefit of patients when this mucoadhesive gel will be available on the market. These findings are protected by a proprietary patent [51].

## Figures and Tables

**Table 1:** Cosmetic ingredients (INCI) included in the basic lipogel used in this work.

Commercial name	Source	INCI	%
LABRAFAC® caprylic/capric MCT (m*edium-chain triglycerides*)	Gattefossè	Caprylic/capric triglyceride	70.0
Ethylcellulose	Asha Cellulose	Ethylcellulose	5.0
COMPRITOL® 888 CG	Gattefossè	Glyceryl behenate	2.5
AEROSIL® R 972	Evonik	Silica dimethyl silylate	3.1
BENECEL™ K4M	Ashland	Hydroxypropyl methylcellulose	11.7
BENECEL™ K100 M	Ashland	Hydroxypropyl methylcellulose	7.0
*Other natural ingredients*			
Aloe vera gel powder regular 200X	Terry Laboratories LLC	*Aloe barbadensis* leaf extract	0.2
Freeze-dried powder blueberries extract, 15% anthocyanins	LaBioTRE	*Vaccinium myrtillus* fruit extract	0.4
Lemon essential oil	Muller and Koster	Citrus limonum peel oil	0.1

**Table 2:** Lyophilized mixture of probiotics enumerated through colony-forming units (CFUs) and Lacto-Counter Assay (LCA).

	Species	Strains	CFUs/g	Bacteria/g; LCA
AL0006 (Lyophilized)	*L. helveticus*	*SP27*	1.6 ± 0.3 × 10^10^	1.2 ± 0.1 × 10^10^
	*L. rhamnosus*	*SP1*		
AL0007 (Lyophilized)	*L. helveticus*	*SP27*	1.0 ± 0.7 × 10^10^	1.0 ± 0.1 × 10^10^
	*L. rhamnosus*	*SP1*		
	*L. paracasei*	*CBA-L87*		

**Table 3:** Comparison of the results obtained with the filter paper test (semiquantitative assessment) and the plate-spreading on the agar test (Quantitative assessment) to estimate the release of bacteria in two different diluents (MRD or SS), and % recovery.

Gel	Diluent	Time points (h)	Plate-spreading Log10 CFUs	Filter paper test Log10 CFUs	% Recovery
AL0006	MRD	T0	9.8	9.7	99.6
	SS		9.5	9.5	99.9
AL0007	MRD	T0	9.5	9.5	100.3
	SS		9.4	9.4	100.0
AL0006	MRD	T2	9.8	9.7	98.9
	SS		9.7	9.5	97.6
AL0007	MRD	T2	9.5	9.5	99.7
	SS		9.4	9.3	99.5
AL0006	MRD	T5	9.6	9.7	101.0
	SS		9.6	9.3	97.3
AL0007	MRD	T5	9.4	9.4	99.9
	SS		9.4	9.2	97.6

**Table 4:** Bacteria released from lipogel samples, incubated in MRD or SS, and the calculated % of adhesion to hydroxyapatite disks.

Gel	Diluent	Time points (h)	3D inset		T0		Adhesion %
			CFUs/ml	Log10 CFUs	CFUs/ml	Log10 CFUs	
AL0006	MRD	T2	6.4*E* + 04	4.8	4.5*E* + 06	6.7	72.2
	SS		1.8*E* + 04	4.3	7.3*E* + 06	6.9	62.1
AL0007	MRD	T2	4.5*E* + 04	4.7	4.3*E* + 06	6.6	70.2
	SS		1.9*E* + 04	4.3	2.5*E* + 06	6.4	67.0
AL0006	MRD	T5	7.3*E* + 04	4.9	4.5*E* + 06	6.7	73.0
	SS		3.0*E* + 04	4.5	7.3*E* + 06	6.9	65.3
AL0007	MRD	T5	2.3*E* + 04	4.4	4.3*E* + 06	6.6	65.8
	SS		6.4*E* + 04	4.8	2.5*E* + 06	6.4	75.2

**Table 5:** Lacto-Counter Assay (LCA) enumeration of bacterial load contained in the two lipogels evaluated at T0 and after 3 (T3), 6 (T6), 9 (T9), and 12 (T12) months on a shelf at room temperature.

Code	T0 (bacteria/g)	T3 (bacteria/g)	T6 (bacteria/g)	T9 (bacteria/g)	T12 (bacteria/g)
AL0006	4.9 ± 0.1 × 10^9^	4.0 ± 0.1 × 10^9^	4.1 ± 0.1 × 10^9^	2.2 ± 0.1 × 10^9^	2.1 ± 0.1 × 10^9^
AL0007	3.9 ± 0.1 × 10^9^	4.3 ± 0.1 × 10^9^	3.8 ± 0.1 × 10^9^	2.5 ± 0.1 × 10^9^	2.1 ± 0.1 × 10^9^

**Table 6:** Lacto-Counter Assay (LCA) enumeration of lactobacilli contained in the lipogels AL0006 and AL0007.

Time (months)	T0	30 min		2 h		5 h		8 h	
	Start	Gel	Solution	Gel	Solution	Gel	Solution	Gel	Solution
AL0006 lipogel preparation									
T0	4.90 × 10^9^	3.81 × 10^9^	2.24 × 10^6^	2.49 × 10^8^	1.55 × 10^7^	1.10 × 10^8^	4.50 × 10^7^	0.68 × 10^8^	6.42 × 10^8^
T3	4.00 × 10^9^	3.70 × 10^9^	5.53 × 10^6^	2.14 × 10^8^	8.63 × 10^6^	1.84 × 10^8^	2.00 × 10^7^	1.13 × 10^8^	2.00 × 10^8^
T6	4.10 × 10^9^	3.23 × 10^9^	3.00 × 10^6^	4.83 × 10^8^	1.44 × 10^7^	4.64 × 10^8^	3.30 × 10^7^	1.19 × 10^8^	8.65 × 10^8^
T9	2.20 × 10^9^	1.97 × 10^9^	6.33 × 10^6^	1.13 × 10^9^	7.23 × 10^6^	6.25 × 10^8^	3.20 × 10^7^	5.77 × 10^7^	7.98 × 10^8^
T12	2.10 × 10^9^	1.97 × 10^9^	6.62 × 10^6^	1.20 × 10^9^	9.46 × 10^6^	6.45 × 10^8^	7.20 × 10^7^	2.35 × 10^7^	1.31 × 10^8^

Time (months)	T0	30 min		2 h		5 h		8 h	
	1 g	1 g	1 ml PS	1 g	1 ml PS	1 g	1 ml PS	1 g	1 ml PS
AL0007 lipogel preparation									
T0	3.90 × 10^9^	3.14 × 10^9^	1.60 × 10^6^	2.60 × 10^8^	1.87 × 10^7^	1.70 × 10^8^	3.80 × 10^8^	1.16 × 10^8^	7.51 × 10^8^
T3	4.30 × 10^9^	2.00 × 10^9^	4.67 × 10^6^	7.60 × 10^8^	1.80 × 10^8^	4.90 × 10^8^	2.70 × 10^8^	1.44 × 10^8^	7.00 × 10^8^
T6	3.80 × 10^9^	1.32 × 10^9^	3.56 × 10^6^	8.04 × 10^8^	2.49 × 10^7^	5.46 × 10^8^	7.34 × 10^8^	2.41 × 10^8^	8.56 × 10^8^
T9	2.50 × 10^9^	1.64 × 10^9^	3.64 × 10^7^	1.50 × 10^9^	4.00 × 10^7^	6.44 × 10^8^	1.83 × 10^8^	1.40 × 10^8^	8.17 × 10^8^
T12	2.12 × 10^9^	1.98 × 10^9^	2.62 × 10^7^	1.10 × 10^9^	8.57 × 10^7^	5.33 × 10^8^	3.12 × 10^8^	9.36 × 10^7^	1.04 × 10^9^

Enumerations of bacteria on lipogels AL0006 (a) and AL0007 (b) and their release in physiological solution. Time T0 is referred to the quantity of lactobacilli present in the lipogels, while time points 30 min, 2, 5, 8 h refer to the quantity of bacteria remaining in the 1 g lipogel (Gel) or released in solutions (1 g/ml) (solution) after each incubation time. Bacterial load and release from lipogels to physiological solution (PS) were evaluated after 3 (T3), 6 (T6), 9 (T9), and 12 (T12) months on a shelf at room temperature. Values are reported as mean. The standard deviation (SD) ranged between 0.1 and 0.3 for all the means and was not reported.

**Table 7:** Results of inhibition zone measures for each pathogen against the three *Lactobacillus* strains selected for lipogel preparations.

Strain	*P. melaninogenica.* DSM 7089	*P. gingivalis* DSM 20709	*A. actinomycetemcomitans* ATCC 700685	*T. forsythia* ATCC 43037	*T. denticola* DSM 14222
*L. rhamnosus SP1*	4.5 ± 0.5	7.0 ± 1	6.5 ± 0.5	7.0 ± 1	7.5 ± 0.5
*L. helveticus SP27*	0	0	3.5 ± 0.5	0	0
*L. paracasei CBA-L87*	8 ± 0.5	10.5 ± 0.5	8.5 ± 0.5	10.5 ± 0.5	8.5 ± 1.5

Note. The data are the mean and SD value of two independent experiments.

**Table 8:** Results of agar diffusion well assay to assess antibacterial properties of the lipogel-bacteria preparations.

Tested conditions	Pathogens blend	*P. melaninogenica* DSM 7089	*P. gingivalis* DSM 20709	*A. actinomycetemcomitans* ATCC 700685	*T. forsythia* ATCC 43037	*T. denticola* DSM 14222
IR0071 (AL0005)	—	—	++	+	++	++
IR0071A (AL0006)	+	+	++	+	++	++
IR0071B (AL0007)	—	—	++	++	++	++
Chlorhexidine 0.2%	+++	+++	+++	+++	+++	+++
MRS broth	—	—	—	—	—	—

Note. All the results were obtained in duplicate. Samples solubilized in SS. “+” indicate a weak inhibition; “++” intermediate inhibition; “+++” strong inhibition; “—” no inhibition.

## Data Availability

All experimental data associated to this study have been included and detailed in this manuscript and in the S. I. There are no further data available. Study of efficacy in a ligature-induced periodontitis mouse model: the study plan and possible amendments, raw data, final report, and other documents pertinent to the study were stored in the Archives of Takis and Alfasigma. In vivo study code: Study AS-Takis 20/C/n.76 as n.64.

## Abbreviations

AL000x: Lab code
CFUs: Colony-forming units
LCA: Lacto-Counter Assay
PBS: Phosphate-buffered saline
SS: Simulated saliva
MDR: Multiple drug resistance
MRD: Maximum recovery diluent
MPs: Modified silicas
HPC: Hydroxypropyl cellulose
MRS: (de Man, Rogosa, and Sharpe) agar
TNBS: 2,4,6-Trinitrobenzene sulfonic acid
CEJ-ABC: Cementoenamel junction-alveolar bone crest
TCP-HA: Tricalcium phosphate/hydroxyapatite.

## Supplementary Materials

Supplementary Materials S.I. Figure 1: Effect of gingival gel treatment (gel AL0006) on the kinetics of total bone loss in the ligated buccal side versus contralateral unligated side at 8, 15, 22, and 29 days after the placement of ligatures on the left maxillary molars in mice. The graphs show measurements on the buccal surfaces indicating bone loss in the ligated side relatively to unligated baseline control. Data shown represent the average + SE of 3 mice/group for sacrifice at 8, 15, and 22 days and 6 mice/group for sacrifice at 29 days. ^*∗*^*P* < 0.05 vs untreated; ^§^*P* < 0.05 vs gel blank (AL0005).
