# Supplementary material for: Probiotics-Containing Mucoadhesive Gel for Targeting the Dysbiosis Associated with Periodontal Diseases
Source: Int J Dent. 2022 Feb 27;2022:5007930. doi: 10.1155/2022/5007930 (PMC8898886; doi:10.1155/2022/5007930)
Supplement: Supplementary Materials — S.I. Figure 1: Effect of gingival gel treatment (gel AL0006) on the kinetics of total bone loss in the ligated buccal side versus contralateral unligated side at 8, 15, 22, and 29 days after the placement of ligatures on the left maxillary molars in mice. The graphs show measurements on the buccal surfaces indicating bone loss in the ligated side relatively to unligated baseline control. Data shown represent the average + SE of 3 mice/group for sacrifice at 8, 15, and 22 days and 6 mice/group for sacrifice at 29 days. ∗P < 0.05 vs untreated; §P < 0.05 vs gel blank (AL0005). [file 5007930.f1.docx]

**SUPPORTING INFORMATION**

In the graph (SI Fig. 1) effect of gingival gels treatment (**AL0006**) on kinetic of total bone loss in ligated buccal side vs contralateral unligated side, at 8, 15, 22 and 29 days after the placement of ligatures on the left maxillary molars in mice.

It is possible to appreciate the effect of the gel **AL0006** vs the reference gel **AL0005**, without probiotics.

In the first week of treatment all groups showed bone loss in the range 0.06-0.12 mm but, from the second week an overall recovery was observe, which continued until day 29. To note the significant bone recovery in group treated with AL0006. This agrees with the fact that in this group no animal has lost the treated (ligated) tooth.

**
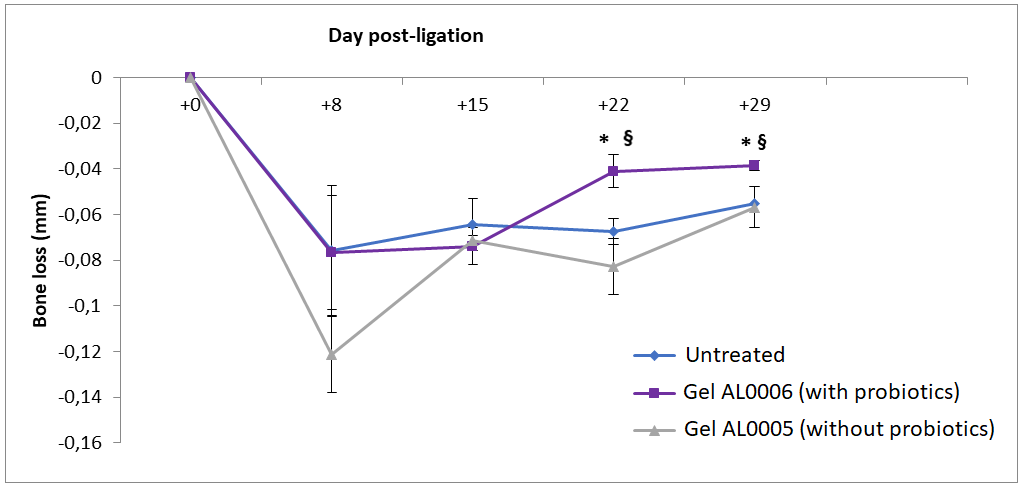
**

**S.I. FIGURE 1**: *Effect of gingival gels treatment (gel* ***AL0006****) on kinetic of total bone loss in ligated buccal side vs controlateral unligated side at 8, 15, 22 and 29 days after the placement of ligatures on the left maxillary molars in mice. The graphs show measurements on the buccal surfaces indicating bone loss in ligated side relatively to unligated baseline control. Data shown represent the average + E.S of 3 mice/group for sacrifices at 8, 15, 22 days and 6 mice/group for sacrifices at 29 days. * P<0.05 vs Untreated; § P<0.05 vs gel Blank (****AL0005****)*
